# A multi-platform analysis of e-cigarette online marketing in China (2024–2025)

**DOI:** 10.1016/j.dialog.2026.100311

**Published:** 2026-05-25

**Authors:** Ruiran Liu, Biaowen Huang, Zheng Wu, Zi Xi, Jidong Huang, Mengjie Guo, Lin Xiao

**Affiliations:** a Tobacco Control Office, Chinese Center for Disease Control and Prevention (Chinese Academy of Preventive Medicine), Beijing, Beijing, China; b School of Language and Communication Studies, Beijing Jiaotong University, Beijing, Beijing, China; c School of Journalism and Communication, Sun Yat-sen University, Guangzhou, Guangdong, China; d Department of Health Policy and Behavioral Sciences, School of Public Health, Georgia State University, Atlanta, GA, USA

**Keywords:** E-cigarettes, Marketing, Current situation

## Abstract

Electronic cigarette (e-cigarette) marketing drives product uptake and use. As internet infrastructure has advanced and smartphone adoption has become widespread, enterprises and merchants have increasingly leveraged online platforms to conduct diverse e-cigarette marketing campaigns. This study aim to characterize the platforms, tactics, and other content features of e-cigarette marketing across Chinese internet platforms and to provide evidence-based recommendations for strengthening regulatory oversight. From September 2024 to January 2025, marketing instances were collected from seven platforms: Xiaohongshu, Douyin, Bilibili, WeChat, Weibo, News websites/APPs, and Online Forums. A China-specific codebook was developed, and the DeepSeek large language model provided auxiliary coding support. Descriptive analyses were performed using frequencies, proportions, and rates, while chi-square tests evaluated differences in tactic selection and publisher distribution across platforms. Among 3596 marketing instances, Xiaohongshu accounted for the largest share (*n* = 2496, 69.4%), followed by Douyin (*n* = 508, 14.1%) and Bilibili (*n* = 318, 8.8%). Direct marketing tactics (*n* = 2164, 60.2%) outnumbered indirect marketing tactics (*n* = 1432, 39.8%). Individual sellers constituted the most prevalent publisher type (*n* = 2604, 72.4%), whereas enterprises were the least common (*n* = 18, 0.5%). Product description (*n* = 1033, 39.7%) and community marketing (*n* = 743, 28.5%) were the two most frequently adopted tactics among individual sellers. Marketing tactic selection differed significantly across platforms (χ^2^ = 1283.8, *P* < 0.001). Despite China's ban on online e-cigarette advertising, this study reveals that publishers continue to employ product descriptions to attract users and leverage community marketing as a covert promotional strategy. Regulators urgently require enhanced monitoring tools to address these increasingly diversified and subtle tactics.

## 1 Introduction

Global e-cigarette use has risen sharply in recent years, with particularly alarming trends observed among adolescents [1]. By 2024, 83 countries had implemented national population-based surveys to assess e-cigarette use among adults – typically those aged 15 years and older, though age ranges vary by survey, while with the rising popularity of e-cigarettes among youth, 118 countries are now monitoring adolescent use through national school-based surveys [2]. Current surveillance data reveal alarming prevalence rates among youth in some countries, such as in Monaco (41%), Lithuania (31%) and Poland (30%) [3].

Mounting evidence confirms that e-cigarettes pose some health risks across multiple physiological systems. These risks include damage to the oral mucosa [4], the development of respiratory diseases [5], and the acceleration of atherosclerosis [6]. Beyond these immediate health concerns, the nicotine content in e-cigarettes creates powerful addiction pathways and impairs critical brain development processes in adolescents [7]. Perhaps most troubling, epidemiological research demonstrates that e-cigarette users, particularly young people who exclusively use these products, face nearly three times the risk of transitioning to traditional cigarette use [8]. Collectively, these findings have established e-cigarette regulation as a central priority within contemporary global tobacco control efforts.

However, sophisticated marketing strategies, particularly those targeting young demographics, present formidable challenges to effective tobacco control implementation [9]. Extensive research across multiple countries has consistently demonstrated that exposure to e-cigarette advertising significantly increases both product uptake and sustained usage rates [10], [11], [12], [13], [14], [15], [16].

China's e-cigarette usage patterns have followed a distinctive trajectory characterized by initial growth followed by decline. Among adolescents, usage rates climbed steadily from 2014 through 2021 [17], [18], [19] before declining through 2023 [20]. Similarly, adult usage demonstrated an upward trend from 2015 to 2018 [21], followed by a notable decrease through 2024 [22]. This reversal can be largely attributed to comprehensive e-cigarette advertising regulations, including the 2022 Measures for the Management of Electronic Cigarettes, which explicitly extended tobacco advertising prohibitions to e-cigarettes [23], and the 2023 Measures for the Administration of Internet Advertising, which further banned online e-cigarette marketing [24].

Although China has made progress in regulating e-cigarettes, the inherent complexity of the online environment has prompted marketers to continually develop increasingly covert promotional methods, thereby complicating regulatory oversight and enforcement. Two Chinese studies examining e-cigarette marketing on social media platforms corroborate this observation. Zhou Xinyi et al. [25]found that posts published by e-cigarette companies on Weibo employing the marketing strategie that downplayed health concerns, only 2.8% posts provided health warnings. Similarly, Ji et al. [26] analyzed e-cigarette-related marketing posts on Xiaohongshu and identified content suggesting that quitting e-cigarettes is difficult, which may serve to indirectly promote continued product use. Collectively, these studies demonstrate that the marketing methods currently deployed by publishers are becoming increasingly subtle and difficult to detect.

International research on online e-cigarette marketing commenced relatively early, with studies primarily analyzing marketing activities on specific websites or social media platforms. A systematic review by Lauren et al. found that social media serves as a popular channel for e-cigarette retailers, who employ diverse strategies to capture the attention of young consumers [27]. By contrast, research on e-cigarette marketing in China has largely concentrated on the sales performance and promotional strategies of e-cigarette companies or e-commerce platforms. Studies that specifically examine the landscape of e-cigarette marketing across social media platforms remain scarce, and systematic approaches to collecting and categorizing marketing content are lacking. Compared with the methodological rigor established in international studies, Chinese research methodologies in this domain remain underdeveloped, with no unified framework for content classification.

To address these regulatory challenges and provide a more comprehensive characterization of e-cigarette marketing in China, this study collected e-cigarette marketing instances from the internet, developed a localized codebook, and employed DeepSeek-assisted coding to systematically describe the volume of marketing instances across different platforms, the marketing tactics employed by various publishers, and additional content characteristics, thereby providing evidence-based recommendations for strengthening tobacco control policies and enhancing regulatory effectiveness.

## 2 Methodology

### 2.1 Data source

Prior to data collection, from June to August 2024, the research team conducted a comprehensive exploratory investigation of e-cigarette marketing practices and consulted the internationally comparable monitoring plan developed by the Tobacco Enforcement and Reporting Movement (TERM) team to establish a keyword list for systematic data extraction (see Online Supplementary Table S1).

Platform selection followed a three-step process. First, drawing on the results of a comprehensive monitoring of e-cigarette marketing activities across all platforms conducted by Beijing Jiaotong University from June to August 2024, we identified representative mainstream Chinese new media platforms. Second, we cross-referenced these platforms with the social media platforms monitored by TERM member countries. Third, the final set of primary platforms for monitoring in China was determined as follows: Weibo (comparable to Twitter), Xiaohongshu (comparable to Instagram), Douyin (comparable to TikTok), Bilibili (comparable to YouTube), WeChat (comparable to Facebook), News websites/APPs, and Online Forums.

Using the established keyword list, Python-based web scrapers were deployed to systematically collect e-cigarette marketing instances from seven distinct platforms between September 2024 and January 2025. Platform access was achieved through automated web scraping (Selenium for dynamic content, BeautifulSoup for static pages), relying exclusively on publicly accessible content. Data collection was conducted monthly (last week of each month) to ensure completeness. The unit of analysis was each individual post (“marketing instance”), including text, images/videos, hashtags, and communication effectiveness metrics. Both organic content and suspected bot accounts/paid advertisements were retained as they represent different marketing tactics. Inclusion criteria: content related to e-cigarette marketing, published on targeted platforms, containing text/images/videos. Exclusion criteria: content explicitly opposing e-cigarette use, duplicates, and unrelated content.

### 2.2 Measures

We constructed a preliminary indicator framework for e-cigarette marketing research on the internet by drawing on multiple sources. These included a comprehensive literature review, the TERM team's codebook for online tobacco marketing research, the implementation guidelines for Article 13 of the WHO Framework Convention on Tobacco Control (“Prohibition of Tobacco Advertising, Promotion, and Sponsorship”) [28], the 2021 World Health Organization Report on the Global Tobacco Epidemic [29], the “5 W” communication theory model [30], and scraped marketing instances. We subsequently conducted a Delphi expert consultation to refine the indicator framework, yielding the final localized codebook (see Online Supplementary Table S2) for analyzing e-cigarette marketing within the Chinese digital landscape.

Qualitative content analysis was performed using the DeepSeek large language model (version DeepSeek-V3.2, developed by Hangzhou DeepSeek Artificial Intelligence Basic Technology Research Co., Ltd.) to provide auxiliary coding support and enhance classification efficiency. Descriptive statistical analyses were then conducted to characterize key dimensions, including marketing platforms, marketing tactics, and other content features. Chi-square tests were employed to evaluate differences in marketing tactic selection and publisher distribution across the various platforms.

### 2.3 Data cleaning and analysis

After removing duplicates (i.e., marketing instances sharing the same author, publication date, link, title, and text) and excluding instances unrelated to e-cigarettes or marketing, a total of 3596 marketing instances remained for analysis. The main coding variables (derived from the indicator framework) used to classify the marketing instances in this paper, along with their definitions, are detailed in Table 1.

**Table 1:** E-cigarette marketing research coding variables.

Variables	Definitions
1 Publisher	The types of accounts that publish marketing information on the internet platform.
1.1 Enterprise	Primarily aimed at making profits and promoting products, it provides goods or services to the market, operates independently, bears its own profits and losses, and is a legal person or a social economic organization. This study includes e-cigarette companies, cultural communication companies, technology companies, and other types of companies (involving e-commerce, retail trade, etc.).
1.2 Individual Seller	The main purpose is to sell products, including individual dealers or agents.
1.3 Ordinary consumer	This includes both members of the general public who have consumed e-cigarettes and those who do not specifically post content related to e-cigarettes.
1.4 Key Opinion Leader (KOL) / Key Opinion Consumer (KOC)	A KOL refers to an individual who holds professional authority or significant influence in a certain field and is accepted or trusted by the relevant group, as well as having considerable influence on the purchasing behavior of that group [31]. A KOC refers to a consumer who can influence their friends, followers, and generate consumption behavior. They achieve this by influencing their private social circle and thereby generating consumption potential [32]. In this study, it is required that the number of fans or followers be greater than 1000, or that they be certified by the platform as having a relatively high level of influence.
1.5 Media	The institutional identifier for disseminating information, data, and content. This study includes official media and general media. Among them, official media refers to media that are operated by the government or have an official certification background.
2 Marketing Platform	The channels through which authors publish marketing information on the Internet.
2.1 WeChat	A mobile platform that integrates instant messaging, social networking, payments, and content sharing.
2.2 Weibo	A platform for sharing and disseminating information based on user relationships, supporting the publication of various types of content.
2.3 Xiaohongshu	A content community platform centered on lifestyle sharing, where users share their life experiences through text, images, and short videos. With its unique focus on “product recommendations” and “reviews,” combined with e-commerce features, the platform has become an important reference for brand promotion and consumer decision-making.
2.4 News websites/APPs	An online platform or mobile application that provides news and information services via the internet. This study includes Southern Metropolis Daily, Baidu Youjia, Top News, East Money, Ordos Online, Guangzhou Daily New Flower City, Hexun, Toutiao, Southern Plus App, Shenzhen Press, Shenzhen News Network, NetEase, Sina, and Tobacco Online.
2.5 Online Forums	Online communities centered around specific topics or interests, where users can post, reply, discuss, and share opinions and information. This study includes Baidu Tieba, Gu Ba, Snowball, and Zhihu.
2.6 Douyin	A social media platform centered around short-form video creation, where users can shoot and edit short videos to share aspects of their lives, talents, and knowledge.
2.7 Bilibili	A long-form video community platform that originated in anime and manga culture and has gradually expanded to include content such as entertainment, knowledge sharing, and lifestyle vlogs.
3 Marketing Tactic	Commercial advertising, promotional activities, or other strategies employed to directly or indirectly market e-cigarettes or encourage their use.
3.1 Direct Marketing	A traditional, direct form of promotion—such as conveying product information to consumers through advertisements, promotional campaigns, and product descriptions—that may serve to directly encourage e-cigarette use.
3.1.1 Direct Brand Advertising	Directly promoting the sale or use of e-cigarette products in a manner that prominently displays product, brand names and logos, providing only the most concise marketing descriptions in both the main text and titles.
3.1.2 Product Description	Describe the product, including detailed explanations of its functions and features, comparisons highlighting the unique characteristics of e-cigarettes versus traditional cigarettes, sharing user experiences, teaching or demonstrating how to authenticate the product and perform maintenance/repairs, and cleaning the e-cigarette.
3.1.3 Price Promotion	Sales strategies utilizing price discounts, complimentary trials, and promotional gifts to stimulate e-cigarette purchases.
3.2 Indirect Marketing	The purpose is not to directly facilitate product purchases. Instead, it involves indirect promotion of e-cigarette companies or brands through soft article placements, promoting non-tobacco products from e-cigarette companies, and disseminating industry news to enhance the company's image and implant brand information in the public's subconscious.
3.2.1 Brand Sponsorship and Public Relations (PR)	Provide financial support for a certain activity, event, or organization, or highlight the achievements of the enterprise to enhance the brand/company image. This can include showcasing spokespersons, sponsoring sports events and concerts, and conducting brand promotion during festivals or commemorative activities.
3.2.2 Corporate Social Responsibility (CSR) and Environmental, Social, Governance (ESG)	Corporate social responsibility projects and ESG ratings displayed to enhance company reputation. ESG represents Environmental, Social, and Governance standards used to evaluate corporate performance across these three dimensions. It also includes providing financial or material assistance to organizations such as community, health, welfare, or environmental protection organizations.
3.2.3 Community Marketing	By connecting brands with specific communities or interest groups, they share industry news, explain usage bans and restrictions, seek product recommendations, and post job openings for e-cigarette retail stores. These circles include hobbyist groups, sports teams, etc.
3.2.4 Brand Extension	Non-tobacco product brands registered by tobacco companies, featuring product packaging that does not evoke associations with tobacco products.
3.2.5 Alternative Sale	Tobacco companies advertising unregulated non-tobacco products to promote regulated tobacco products, utilizing packaging designs that evoke tobacco product associations.
3.2.6 Content Implantation	The titles and main text are unrelated to e-cigarette marketing, but the hashtags are e-cigarette-related, creating a general marketing effect; or the titles, main text, and hashtags are all unrelated to e-cigarette marketing, yet e-cigarette-related images or video are displayed.
4 Brand and Product Type	*E*-cigarette brands and types mentioned in marketing instances.
5 E-cigarette flavor	The Marketing Situation of E-cigarette Flavors as Illustrated by Marketing Instances.
5.1 Specifically mentions flavors	A special feature on one or more e-cigarette flavors.
5.2 Generally refers to flavors	A general reference to e-cigarette flavors without introduction.
5.3 Does not mention flavors	There was no mention of e-cigarette flavors.
6 Sentiment	The views on e-cigarettes as reflected in the marketing instances.
6.1 Positive	Holds a positive stance toward e-cigarettes, supporting their consumption and use.
6.2 Negative	Holds a negative stance toward e-cigarettes, emphasizing their harms and the need for regulation.
6.3 Neutral	Holds a neutral stance or has no clearly defined position toward e-cigarettes.
7 Communication Effectiveness	The impact of marketing content on audiences' psychological, attitudinal, and behavioral dimensions is reflected through quantitative metrics such as shares, comments, likes, reads, and collections.

To automate the coding process, we developed a supervised machine learning system for classifying e-cigarette marketing instances. While maintaining a consistent large language model (LLM) architecture, we optimized performance by testing multiple prompt configurations and incorporating Retrieval-Augmented Generation (RAG) to enhance coding accuracy, building a Knowledge Base (Example Database + Rule Base based on the codebook). The entire approach was implemented using Python programming, and the complete workflow is detailed in Online Supplementary Fig. S1.

First, the marketing data was split into a prediction set (unlabeled) and a sample set (labeled) in a 9:1 ratio. Second, for the sample set, stratified sampling was performed based on the proportion of marketing volume across each platform; a certain amount of data was extracted from each platform, and the remainder was added to the prediction set. The sample set was manually coded by one trained coder and reviewed by tobacco control media experts at Beijing Jiaotong University; Finally, the sample set underwent 10-fold cross-validation, where it is randomly divided into 10 equal parts: 9 parts served as the training set to build the example database within the knowledge base, and the remaining 1 part served as the validation set.

The prompts were iteratively refined based on the DeepSeek encoding results and reasoning processes obtained from each prompt configuration, ultimately yielding five distinct prompt-DeepSeek encoding combinations. Each combination underwent 10 rounds of machine coding via 10-fold cross-validation. In each round, 9 randomly selected data points from the training set built the knowledge base, while the remaining data point served as the validation set for performance evaluation. The optimal prompt-model combination was identified by comparing average performance across all prompts. Because this study equally prioritized true positive and true negative classifications, accuracy served as the only performance metric. The fifth prompt achieved the highest accuracy (91.17%) and was therefore selected for final implementation (model accuracy for each prompt set is presented in Online Supplementary Table S3; the fifth prompt set is available in Online Supplementary Text S1). This optimized model was then applied to encode the entire prediction set using the knowledge base constructed from all sample sets. Once machine coding is complete, a manual review is conducted, primarily to verify whether any single-choice options have been mistakenly selected as multiple-choice, and whether any options fall outside the predefined categories. Notably, to reduce the complexity of DeepSeek coding, category assignments were determined based on the most salient features exhibited in each marketing instance. For example, it is coded according to the largest proportion of content.

Data were subsequently analyzed using Microsoft Excel and SAS 9.4. Each coding variable was described using frequencies, rates, or proportions. Chi-square tests were employed to assess differences across marketing platforms in terms of marketing tactic selection and publisher distribution (two-tailed, α = 0.05). To objectively quantify sentiment, engagement metrics were used to calculate sentiment scores based on log-transformed like counts ($\mathrm{score}=\log10\left(\mathrm{likes}+1\right)$) for each marketing instance, thereby reducing skewness [26]. Marketing instances with positive, negative, and neutral sentiment received corresponding positive, negative, and zero scores, respectively. A scatter plot was generated to visualize sentiment polarity, with marketing instances plotted on the horizontal axis and their corresponding sentiment scores on the vertical axis.

This study was exempted from human subjects review because it exclusively involved content analysis of publicly available social media posts and did not include human research participants. No personally identifiable information was collected from post authors, and no names are disclosed in this article. The study adheres to each platform's terms of service and all applicable laws and regulations; accordingly, ethical approval was not required.

## 3 Results

### 3.1 Marketing platforms

**Fig. 1:**
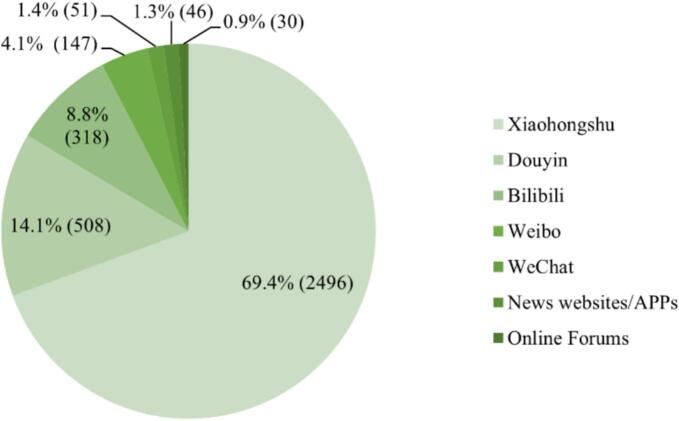
Marketing platforms for all marketing instances (*N* = 3596).

A total of 3596 marketing instances were collected across the seven monitored platform categories. Xiaohongshu demonstrated the highest volume with 2496 instances (69.4%), followed by Douyin with 508 (14.1%), Bilibili with 318 (8.8%), Weibo with 147 (4.1%), WeChat with 51 (1.4%), News websites/APPs with 46 (1.3%), and Online Forums with 30 (0.9%) (see Fig. 1).

### 3.2 Marketing tactics

Direct marketing tactics (60.2%, 2164/3596) substantially outnumbered indirect marketing tactics (39.8%, 1432/3596) across all collected instances. The three most prevalent tactics were product description (39.0%, 1401/3596), community marketing (25.4%, 912/3596), and direct brand advertising (19.9%, 717/3596). Other marketing tactics were marginally represented, with price promotion, brand sponsorship and public relations, corporate social responsibility (CSR) and environmental, social, governance (ESG) initiatives, brand extension, alternative sale, and content implantation each accounting for less than 12.0% of marketing instances. Beyond these aggregate patterns, several qualitative findings merit attention. One marketing instance employed brand sponsorship and PR tactics to showcase how RELX, a leading e-cigarette brand, sponsored the concert tour of Liu Cong, a prominent Chinese singer. Photographs within the post revealed numerous RELX-branded display boards at the venue, exposing young attendees to the brand and potentially fostering interest in e-cigarettes. In a separate pattern, individual sellers strategically disseminated store information across social media platforms, effectively establishing an “online attraction + offline sales” business model.

Different types of publishers employ varying marketing tactics when promoting e-cigarettes. (see Table 2). Individual sellers constituted the dominant publisher category (72.4%, 2604/3596) and predominantly utilized product description tactics (39.7%, 1033/2604) and community marketing tactics (28.5%, 743/2604). Ordinary consumers (24.3%, 874/3596) and Key Opinion Leaders/Key Opinion Consumers (2.3%, 81/3596) similarly employed product description tactics (36.6% and 51.9%, respectively), sharing personal usage experiences to enhance brand credibility and product appeal from individual perspectives. Enterprises (0.5%, 18/3596) primarily utilized product description and CSR initiatives (33.3%, 6/18) to build consumer trust and encourage product adoption. For example, a cultural communications company posted an article introducing RELX's ESG rating, noting that Fogcore Technology is committed to green development and sustainability, which would enhance RELX's public image. Media outlets also predominantly employed CSR and ESG tactics (89.5%, 17/19) to enhance public perception of e-cigarette companies, publishing positive news related to the e-cigarette industry while promoting corporate efforts in technological innovation and social responsibility.

**Table 2:** Marketing tactics by publishers.

	All publishers	Enterprise	Individual seller	Ordinary consumer	KOL/KOC	Media
	*n* (%)	*n* (%)	*n* (%)	*n* (%)	*n* (%)	*n* (%)
Total	3596 (100.0)	18 (0.5)	2604 (72.4)	874 (24.3)	81 (2.3)	19 (0.5)

Marketing tactics						
Direct brand advertising	717 (19.9)	4 (22.2)	482 (18.5)	205 (23.5)	26 (32.1)	0 (0.0)
Price promotion	46 (1.3)	0 (0.0)	34 (1.3)	11 (1.3)	1 (1.2)	0 (0.0)
Product description	1401 (39.0)	6 (33.3)	1033 (39.7)	320 (36.6)	42 (51.9)	0 (0.0)
Brand sponsorship and PR	28 (0.7)	0 (0.0)	16 (0.6)	8 (0.9)	2 (2.4)	2 (10.5)
CSR and ESG	75 (2.1)	6 (33.3)	37 (1.4)	15 (1.7)	0 (0.0)	17 (89.5)
Community marketing	912 (25.4)	2 (11.2)	743 (28.5)	162 (18.5)	5 (6.2)	0 (0.0)
Brand extension	0 (0.0)	0 (0.0)	0 (0.0)	0 (0.0)	0 (0.0)	0 (0.0)
Alternative sale	0 (0.0)	0 (0.0)	0 (0.0)	0 (0.0)	0 (0.0)	0 (0.0)
Content implementation	417 (11.6)	0 (0.0)	259 (10.0)	153 (17.5)	5 (6.2)	0 (0.0)

Chi-square analysis revealed highly significant differences in the distribution of marketing tactics across platforms (χ^2^ = 1283.8, Ρ < 0.001), with each platform exhibiting a distinct tactic profile (Table 3). On WeChat, product description was the dominant strategy (60.8%, 31/51), followed by CSR and ESG (23.5%, 12/51) and direct brand advertising (5.9%, 3/51). On Weibo, direct brand advertising predominated (40.8%, 60/147), followed by product description (17.0%, 25/147) and content implementation (14.3%, 21/147). Xiaohongshu was similarly led by product description (37.5%, 935/2496), with community marketing a close second (34.7%, 866/2496) and direct brand advertising third (15.2%, 380/2496). News websites/apps were distinguished by a high prevalence of CSR and ESG tactic (34.8%, 16/46), followed by direct brand advertising (23.9%, 11/46) and product description (21.7%, 10/46). On Online Forums, community marketing was most common (33.3%, 10/30), followed by product description (26.7%, 8/30) and content implementation (16.6%, 5/30). Douyin was led by direct brand advertising (35.8%, 182/508), followed by product description (32.7%, 166/508) and content implementation (26.7%, 136/508). Finally, Bilibili showed the highest concentration of product description tactic (71.1%, 226/318), with direct brand advertising second (24.8%, 79/318) and community marketing a distant third (1.9%, 6/318).

**Table 3:** Distribution of marketing tactics and publishers across different marketing platforms.

	WeChat	Weibo	Xiaohongshu	News websites/APPs	Online forums	Douyin	Bilibili	χ^2^	Ρ
	*n* (%)	*n* (%)	*n* (%)	*n* (%)	*n* (%)	*n* (%)	*n* (%)		
Marketing tactics								1283.8	<0.001
Direct brand advertising	3 (5.9)	60 (40.8)	380 (15.2)	11 (23.9)	2 (6.7)	182 (35.8)	79 (24.8)		
Price promotion	2 (3.9)	19 (12.9)	12 (0.5)	2 (4.4)	3 (10.0)	4 (0.8)	4 (1.3)		
Product description	31 (60.8)	25 (17.0)	935 (37.5)	10 (21.7)	8 (26.7)	166 (32.7)	226 (71.1)		
Brand sponsorship and PR	1 (2.0)	2 (1.4)	16 (0.6)	3 (6.5)	0 (0.0)	6 (1.2)	0 (0.0)		
CSR and ESG	12 (23.5)	1 (0.7)	36 (1.4)	16 (34.8)	2 (6.7)	6 (1.2)	2 (0.6)		
Community marketing	2 (3.9)	19 (12.9)	866 (34.7)	1 (2.2)	10 (33.3)	8 (1.6)	6 (1.9)		
Brand extension	0 (0.0)	0 (0.0)	0 (0.0)	0 (0.0)	0 (0.0)	0 (0.0)	0 (0.0)		
Alternative sale	0 (0.0)	0 (0.0)	0 (0.0)	0 (0.0)	0 (0.0)	0 (0.0)	0 (0.0)		
Content implementation	0 (0.0)	21 (14.3)	251 (10.1)	3 (6.5)	5 (16.6)	136 (26.7)	1 (0.3)		
Publishers								2708.9	<0.001
Enterprise	16 (31.4)	0 (0.0)	0 (0.0)	0 (0.0)	0 (0.0)	2 (0.4)	0 (0.0)		
Individual seller	21 (41.2)	75 (51.0)	2065 (82.7)	9 (19.6)	8 (26.7)	284 (55.9)	142 (44.7)		
Ordinary consumer	14 (27.4)	56 (38.1)	431 (17.3)	17 (36.9)	18 (59.9)	211 (41.5)	127 (39.9)		
KOL/KOC	0 (0.0)	15 (10.2)	0 (0.0)	4 (8.7)	2 (6.7)	11 (2.2)	49 (15.4)		
Media	0 (0.0)	1 (0.7)	0 (0.0)	16 (34.8)	2 (6.7)	0 (0.0)	0 (0.0)		

Chi-square analysis also revealed highly significant differences in publisher composition across platforms (χ^2^ = 2708.9, Ρ < 0.001) (Table 3). On WeChat, individual sellers constituted the largest publisher group (41.2%, 21/51), followed by enterprises (31.4%, 16/51) and ordinary consumers (27.4%, 14/51). On Weibo, individual sellers were again the most prevalent (51.0%, 75/147), followed by ordinary consumers (38.1%, 56/147) and KOLs/KOCs (10.2%, 15/147). Xiaohongshu showed the strongest concentration of individual sellers of any platform (82.7%, 2065/2496), with ordinary consumers accounting for the remainder (17.3%, 431/2496). News websites/apps diverged markedly from this pattern: ordinary consumers were the most common publishers (36.9%, 17/46), followed by media outlets (34.8%, 16/46) and individual sellers (19.6%, 9/46). Online Forums showed a similar divergence, with ordinary consumers dominant (59.9%, 18/30), followed by individual sellers (26.7%, 8/30) and KOLs/KOCs and media outlets each accounting for 6.7% (2/30). On Douyin, individual sellers were again the leading publisher type (55.9%, 284/508), followed by ordinary consumers (41.5%, 211/508) and KOLs/KOCs (2.2%, 11/508). Bilibili showed a comparable distribution: individual sellers led (44.7%, 142/318), followed by ordinary consumers (39.9%, 127/318) and KOLs/KOCs (15.4%, 49/318).

### 3.3 E-cigarette flavors

**Fig. 2:**
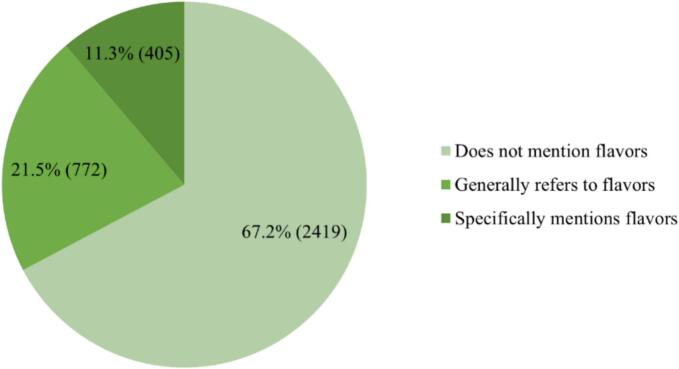
Mentions of e-cigarette flavors (N = 3596).

Despite regulatory prohibitions on non-tobacco flavored e-cigarettes, this study found that 32.8% (1177/3596) of marketing instances still mentioned e-cigarette flavors, while 11.3% (405/3596) provided detailed descriptions of them (Fig. 2). Further analysis revealed that most promoted flavors were named after fruits, desserts, beverages, and other food-related items, enabling audiences to immediately associate each product with familiar, appealing taste profiles.

### 3.4 Communication effectiveness

We quantified overall communication effectiveness by aggregating engagement metrics (likes, shares, comments, collections, and reads) for each marketing instance. The three most effective instances employed distinct tactics across different platforms: A KOL/KOC employs a content implantation tactic: while the titles, main text, and hashtags of the marketing instances bore no relation to e-cigarette marketing, yet e-cigarette products were featured within the entertaining video on Bilibili (32,783 communication effectiveness volumes); RELX product imagery from an individual seller using direct brand advertising tactics to showcase the brand's building block series on Douyin (15,135 communication effectiveness volumes); and an enterprise-published informational article employing product description tactics to introduce products launched in December 2024 on WeChat (6631 communication effectiveness volumes).

### 3.5 E-cigarette brands and product types

RELX (Relx Technology Inc.) dominated brand mentions with an 81.0% (2913/3596) share, followed by other brands (7.6%, 273/3596), non-specific brands (3.6%, 130/3596), SNOWPLUS (3.2%, 116/3596), LAMI (2.0%, 71/3596) (see Online Supplementary Fig. S2). The remaining brands captured minimal market share. Among product types, RELX Infinity 2 exhibited the relatively high representation (20.6%, 740/3596). Additional details are provided in Online Supplementary Fig. S3.

### 3.6 Sentiment

**Fig. 3:**
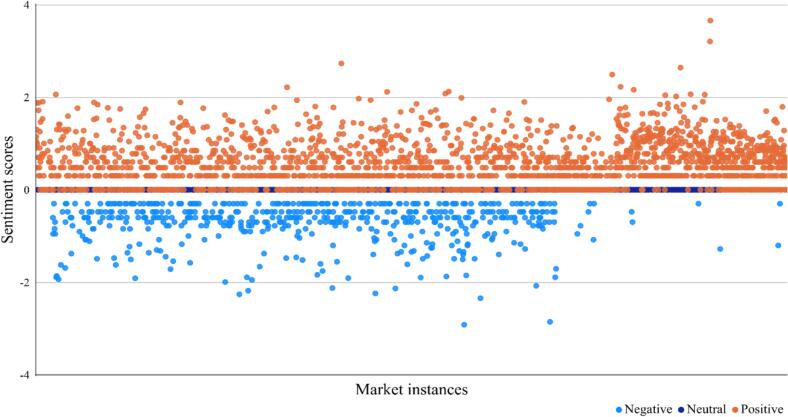
Sentiment distribution of all marketing instances.

Sentiment analysis was conducted to evaluate the attitudinal stance of marketing instances toward e-cigarettes. The sentiment distribution revealed a predominance of positive attitudes toward e-cigarettes (56.1%, 2018/3596), followed by neutral attitudes (24.8%, 890/3596) and negative attitudes (19.1%, 688/3596) (see Online Supplementary Fig. S4). Marketing instances expressing positive attitudes toward e-cigarettes received higher sentiment scores, indicating greater user engagement (see Fig. 3). Notably, some neutral marketing instances expressed mixed sentiments, acknowledging both advantages and disadvantages of e-cigarettes. Representative expressions included statements such as “the appearance is stylish and compact, with a cool color scheme but can affect the development of brain nerves”.

## 4 Discussion

Xiaohongshu emerged as the primary platform for e-cigarette marketing, a finding attributable to its user demographics, which consist primarily of young people in developed regions characterized by substantial income, trend-following behaviors, and elevated platform engagement [33]. The lower volume of marketing instances observed on other platforms may reflect their participation in smoke-free internet initiatives (e.g., Zhihu, Bilibili, Weibo) and more stringent regulatory enforcement [34]. Nevertheless, these platforms collectively form a diverse dissemination landscape alongside Xiaohongshu, and we recommend that Xiaohongshu also participate in smoke-free internet initiatives in the future.

This study found that direct marketing tactics predominated, consistent with findings from a systematic review conducted internationally [35]. Notably, direct brand advertising tactics were employed less frequently than product description approaches, suggesting that stringent Chinese regulations on online advertising have successfully influenced publishers' choices regarding marketing tactics. In addition, community marketing tactics ranked second in this study (25.4%). These tactics involve connecting the brand with specific communities or interest groups to share industry news, explain usage restrictions and regulations, solicit product recommendations, and post job openings at vape retail stores. Creating and sharing content that is both engaging and practical can foster long-term connections between the brand and its target audience, thereby not only enhancing brand awareness and favorability but also strengthening user loyalty [36]. Therefore, regulatory oversight should extend beyond traditional advertising to encompass all tactics that promote e-cigarette use.

Individual sellers emerged as the predominant publishers of e-cigarette marketing in this study, consistent with a Chinese report on online tobacco marketing monitoring, which found that tobacco agents accounted for the vast majority (76.13%) of tobacco advertising and promotional materials [37]. Individual sellers primarily use the product description tactic to highlight features such as packaging design, flavor profiles, battery capacity, and the number of puffs. Notably, individual sellers also strategically leveraged community marketing—their second most frequently employed tactic—by framing discussions around policy restrictions and regulatory bans as opportunities to display e-cigarette imagery, thereby partially circumventing advertising regulations. For example, the main text of a marketing instance indicates: “The government has issued a ban on the sale of RELX e-cigarette products! The government has explicitly prohibited the sale of fruit-flavored e-cigarettes. While some people may still have access to them, be sure to exercise caution and avoid risks before purchasing. Some e-cigarettes laced with drugs appear no different from ordinary products, making it easy to let your guard down. If you experience dizziness, trembling hands, prolonged and intense mental agitation, or a darkening of the complexion after use, you may have accidentally inhaled a harmful e-cigarette. Be sure to identify reputable manufacturers, quit smoking as soon as possible, avoid exposure, and do not cause your family worry”. However, the image of the marketing instance prominently displays a RELX e-cigarette. Future oversight efforts on new media platforms should therefore prioritize developing effective monitoring mechanisms for individual seller accounts. Enterprise accounts represent a very small proportion of publishers, likely attributable to China's strict regulations on online e-cigarette marketing [24], which prevent companies from freely posting promotional content across various platforms. Meanwhile, enterprises tend to utilize CSR and ESG initiatives to divert attention from health risks, as exemplified by IMiracle Technology Co's website detailing environmental, social, and governance achievements that emphasize environmental protection. World Health Organization (WHO) research on tobacco product regulation indicates that e-cigarette manufacturers strategically deploy CSR initiatives to circumvent regulation, enhance public image, and promote brands, as demonstrated by Altria's 2018 charitable donations to the National Museum of African American History and Culture and Boys and Girls Clubs [38]. Notably, we also found that few enterprises continue to employ direct brand advertising tactics. For example, prominently featuring RELX products in the video, but providing only the most concise marketing descriptions in both the main text and titles, underscoring the need for relevant authorities to strengthen oversight of e-cigarette advertising.

This study found that dominant marketing tactics varied considerably across platforms: product description prevailed on WeChat, Xiaohongshu, and Bilibili; direct brand advertising dominated on Weibo and Douyin; CSR and ESG tactic was most prominent on news websites/apps; and community marketing led on online forums. These platform-specific patterns suggest that regulatory measures should be tailored to the distinct marketing characteristics of each platform. This study also found that individual sellers were the predominant publisher type across most platforms—specifically WeChat, Weibo, Xiaohongshu, Douyin, and Bilibili—whereas ordinary consumers constituted the largest publisher group on news websites/apps and online forums. This distinction highlights the need for platform-specific regulatory approaches that account for the differing account types through which e-cigarette marketing content is disseminated.

The 2022 Measures for the Management of Electronic Cigarettes explicitly prohibit the sale of flavored e-cigarettes other than tobacco flavor [23]. Despite this regulation, 11.3% of marketing instances continued to provide detailed descriptions of e-cigarette flavors, promoting e-cigarette flavors other than tobacco to attract customers has kept the market demand for fruit-flavored e-cigarettes alive. Kostygina et al. [39] reported that 40.6% of Instagram posts involved flavor promotion, while Shah et al. [40] found that over half (53.0%) of Instagram posts marketed specific e-cigarette flavors—from mint to numerous others—that appeal to adolescents and young adults. Given that enticing flavors represent the most commonly cited reason for e-cigarette use among young people [41], stricter enforcement is warranted against marketing instances that describe or recommend flavors.

Notably, humorous videos featuring e-cigarette products posted on Bilibili have achieved significant reach, with 32,783 communication effectiveness volumes. Young people are likely to encounter such entertaining content, prompting regulatory authorities to monitor similar content closely and take appropriate enforcement action.

The high frequency of RELX mentions reflects a concentrated media and marketing campaign strategy that establishes the brand as a focal point through repeated dissemination across platforms. This finding aligns with the established 2023 e-cigarette brand hierarchy in China [42].

Sentiment analysis revealed that marketing instances conveyed diverse attitudes toward e-cigarettes, with positive sentiments predominating (56.1%). Positive narratives often highlight the aesthetic design, packaging colors, functional features, and unique flavors of e-cigarettes, while also emphasizing that they are less harmful than traditional cigarettes and can serve as a smoking cessation aid. Negative content, by contrast, centered on product bans, usage restrictions, and health risks. Additionally, some marketing instances expressed ambivalent perspectives—presenting both advantages and disadvantages of e-cigarettes—which may obscure regulatory oversight and enable such content to evade detection.

The application of artificial intelligence to tobacco control research represents a notable methodological development. This study employed DeepSeek for auxiliary coding in e-cigarette marketing surveillance, demonstrating the feasibility of a scalable, AI-assisted monitoring framework. With cross-validation accuracy exceeding 90%, this approach showed sufficient reliability for large-scale content classification tasks. However, further validation across diverse datasets and marketing contexts is needed before such tools can be broadly adopted for regulatory enforcement. Nevertheless, AI-assisted coding offers considerable potential for improving the efficiency and consistency of platform-level surveillance efforts.

## 5 Conclusion

This study systematically characterized the platforms, tactics, and content features of online e-cigarette marketing in China, revealing that contemporary marketing tactics increasingly rely on diversified methods—particularly product descriptions and community marketing—that may evade traditional surveillance and regulatory frameworks. Based on these findings, we offer three specific recommendations. First, regulatory authorities should deploy AI-assisted monitoring systems capable of real-time, cross-platform detection of marketing tactics, including community marketing that ostensibly discusses regulations while displaying product imagery. Second, platform-specific enforcement protocols should be developed, with particular attention to Xiaohongshu, where the highest concentration of marketing activity was observed. Third, social media platforms that have not yet joined smoke-free internet initiatives should be actively recruited to adopt tobacco control agreements and implement robust self-monitoring mechanisms with transparent reporting requirements.

## 6 Limitations

The five-month data collection period of this study failed to capture all potential marketing peaks, including major promotional periods such as the Lantern Festival, Women's Day, Dragon Boat Festival, and World No Tobacco Day. This temporal constraint limits our understanding of seasonal variations in both marketing volume and tactical approaches during key promotional windows, ultimately resulting in incomplete coverage of the full spectrum of marketing activities throughout the year.

DeepSeek represents an LLM specifically engineered for textual data processing and natural language comprehension; however, it lacks the capability to process multimodal content, including images, videos, and audio files. This technical limitation leads to visual elements cannot be accurately analyzed. Furthermore, because we utilized pre-deployed model versions, we were unable to modify model parameters, which constrained our ability to mitigate model hallucinations and optimize performance for this specific application.

Marketing instances from Xiaohongshu accounted for nearly 70% of the dataset. This distributional imbalance may partly reflect differences in platform accessibility and scraping efficiency during data collection. Consequently, caution is warranted when generalizing these findings to other platforms or drawing cross-platform comparisons.

Due to limitations in human and financial resources, the relatively objective indicators in the codebook used in this study—such as “Publisher,” “Marketing Platform,” and “Communication Effectiveness”—were coded by a single coder.

## CRediT authorship contribution statement

**Ruiran Liu:** Writing – original draft, Validation, Software, Methodology, Formal analysis, Data curation, Conceptualization. **Biaowen Huang:** Validation, Software, Methodology, Investigation, Data curation, Conceptualization. **Zheng Wu:** Investigation, Data curation. **Zi Xi:** Conceptualization. **Jidong Huang:** Writing – review & editing, Conceptualization. **Mengjie Guo:** Formal analysis. **Lin Xiao:** Writing – review & editing, Supervision, Project administration, Funding acquisition, Conceptualization.

## Funding

E-cigarette Marketing Monitoring Study.

## Declaration of competing interest

The authors declare that there are no competing financial interests or any personal links that have influenced the data generation of this research paper.
