# Supplementary material for: A multi-platform analysis of e-cigarette online marketing in China (2024–2025)
Source: Dialogues Health. 2026 May 25;8:100311. doi: 10.1016/j.dialog.2026.100311 (PMC13233575; doi:10.1016/j.dialog.2026.100311)
Supplement: Supplementary file 1 — Supplementary material [file mmc1.zip › Table S2 Codebook for Online Marketing of E-cigarettes in China (Excerpt).docx]

Table S2 Codebook for Online Marketing of E-cigarettes in China (Excerpt)

| **Coded entry** | **Notes** |
| --- | --- |
| 1 Post id | The marketing instance numbers, numbered from 1 to n (where n is the total number of marketing instances). Manually sorted. |
| 2 Title | Headlines of marketing instances. Python scraping. |
| 3 Author | The name of the account that posted the content. Python scraping. |
| 4 Publication date | The time the marketing instance was published. Python scraping. |
| 5 Link | A link to a marketing instance. Python scraping. |
| 6 Text | The content of a marketing instance, with hashtags separated from it. Python scraping. |
| 7 Publisher | Types of accounts that post marketing content on internet platforms. 1=Enterprise、2=Individual Seller、3=Ordinary consumer、4=Key Opinion Leader (KOL) / Key Opinion Consumer (KOC)、5=Media。Manually coded. |
| 8 Marketing Platform | Channels through which authors publish marketing information on the internet. 1=WeChat、2=Weibo、3=Xiaohongshu、4=News websites/APPs、5=Online Forums、6=Douyin、7=Bilibili。Manually coded. |
| 9 Marketing content | Marketing messages and content delivery formats. |
| 9.1 Marketing Tactic | Tactics used to promote e-cigarettes, either directly or indirectly. 1=Direct Brand Advertising、2=Price Promotion、3=Product Description、4=Brand Sponsorship and Public Relations (PR)、5=Corporate Social Responsibility (CSR) and Environmental, Social, Governance (ESG)、6=Community Marketing、7=Brand Extension、8=Alternative Sale、9=Content Implantation. Direct brand advertising, price promotions, and product description fall under direct marketing; the rest fall under indirect marketing. AI coded. |
| 9.2 Brand | The e-cigarette brands mentioned in the marketing instance. 1=RELX、2=MOTI、3=LAMI、4=YOOZ、5=SNOWPLUS、6=COEE、7=MEVOL、8=SMOK、9=BOULDER、10=Others、11=Non-specific brands。AI coded. |
| 9.3 Product Type | The e-cigarette models mentioned in the marketing instance. 1=RELX Phantom、2=RELX Artist Series、3=RELX Alpha、4=RELX infinity 2、5=RELX Daqian、6=YOOZ Veex 5、7=MOTI S、8=Feelm Air 4、9=LAMI M、10=SNOWPLUS Platypus、11=Others、12=Non-specific types. AI coded. |
| 9.4 E-cigarette flavor | Marketing of e-cigarette flavors in marketing instances. 1=Specifically mentions flavors, 2=Generally refers to flavors, 3=Does not mention flavors. AI coded. |
| 9.5 Sentiment | Marketing instances reflect attitudes toward e-cigarettes. 1=Positive、2=Negative、3=Neutral。AI coded. |
| 10 Communication Effectiveness | These include five metrics: likes, shares, collections, comments, and reads. Manually coded. |
