# Supplementary material for: A multi-platform analysis of e-cigarette online marketing in China (2024–2025)
Source: Dialogues Health. 2026 May 25;8:100311. doi: 10.1016/j.dialog.2026.100311 (PMC13233575; doi:10.1016/j.dialog.2026.100311)
Supplement: Supplementary file 1 — Supplementary material [file mmc1.zip › Table S3 Model evaluation based on five prompts.docx]

Table S3 Model evaluation based on five prompts

| Results | Accuracy (%) |
| --- | --- |
| Trial 1 | 89.86 |
| Trial 2 | 90.87 |
| Trial 3 | 90.98 |
| Trial 4 | 88.73 |
| Trial 5 | 91.17 |
