# Supplementary material for: A multi-platform analysis of e-cigarette online marketing in China (2024–2025)
Source: Dialogues Health. 2026 May 25;8:100311. doi: 10.1016/j.dialog.2026.100311 (PMC13233575; doi:10.1016/j.dialog.2026.100311)
Supplement: Supplementary file 1 — Supplementary material [file mmc1.zip › Table S1 The keyword list for marketing instances scraping.docx]

Table S1 The keyword list for marketing instances scraping

| **1.** **Another name or jargon for E-cigarettes** | **2.** **E-cigarettes brands** | **3.** **Topics related to e-cigarette marketing** |
| --- | --- | --- |
| Electronic rations (excluding: tobacco control, variety entertainment, games) | Relx/The fifth generation of Phantom/Artist’s pen | Herbal nebulizer / Herbal vaporizer / Electronic inhaler |
| Electronic fire cause (Chinese character (“烟”) splitting two part) | zero |  |
| Electronic atomizer/electronic atomizer equipment | MOTI |  |
| Smoke cartridges/Wildcard cartridges/Smoke balls/Wildcard balls | lami |  |
|  | elfbar |  |
|  | Yooz |  |
|  | VTV |  |
|  | mevol |  |
|  | song |  |
|  | moosee |  |
|  | waka |  |
