# Supplementary figures and images for: A multi-platform analysis of e-cigarette online marketing in China (2024–2025)
Source: Dialogues Health. 2026 May 25;8:100311. doi: 10.1016/j.dialog.2026.100311 (PMC13233575; doi:10.1016/j.dialog.2026.100311)

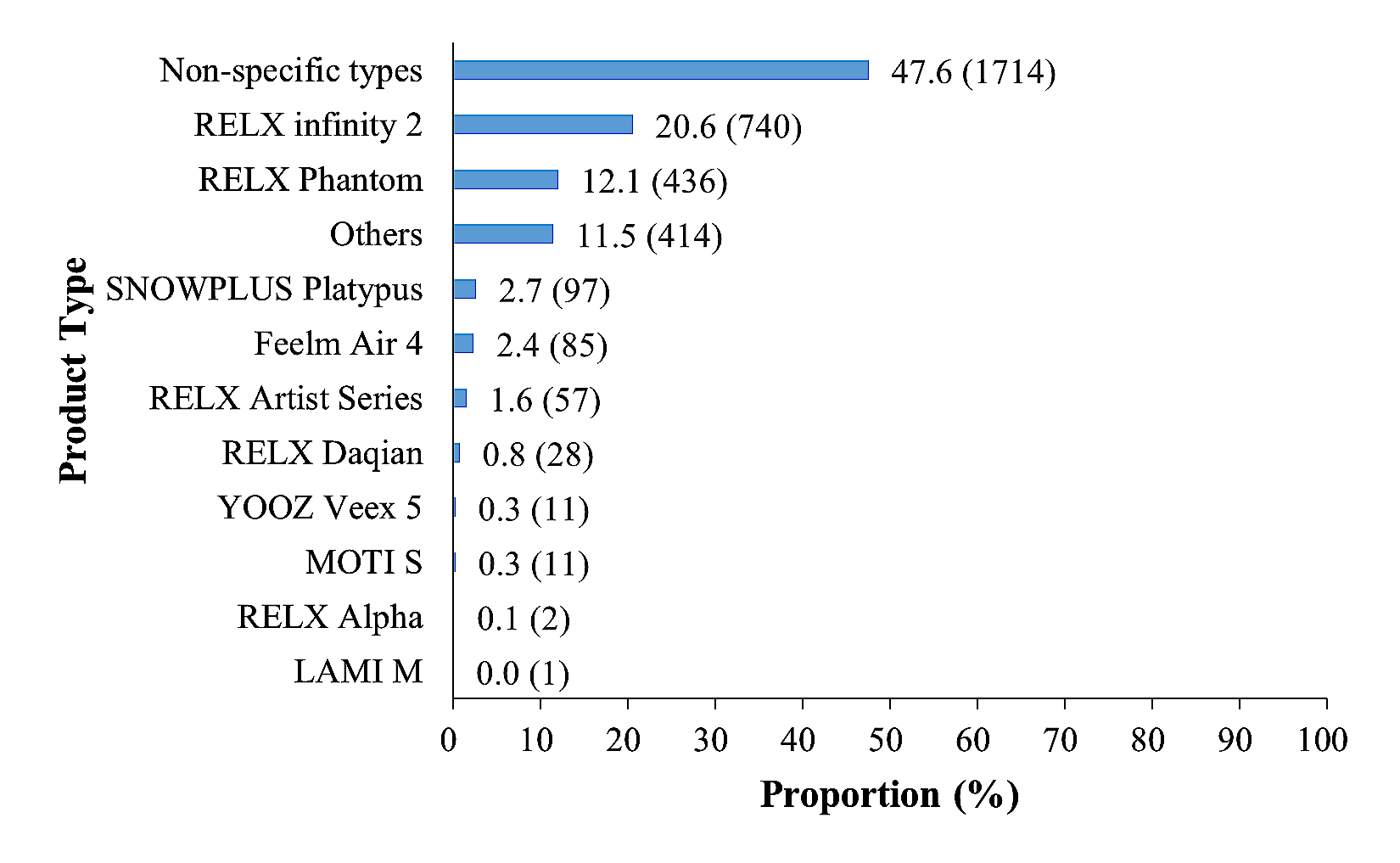

Supplement: Supplementary file 1 — Supplementary material [file mmc1.zip › Figure S3 Proportion of e-cigarette product type mentions (N=3,596).png]

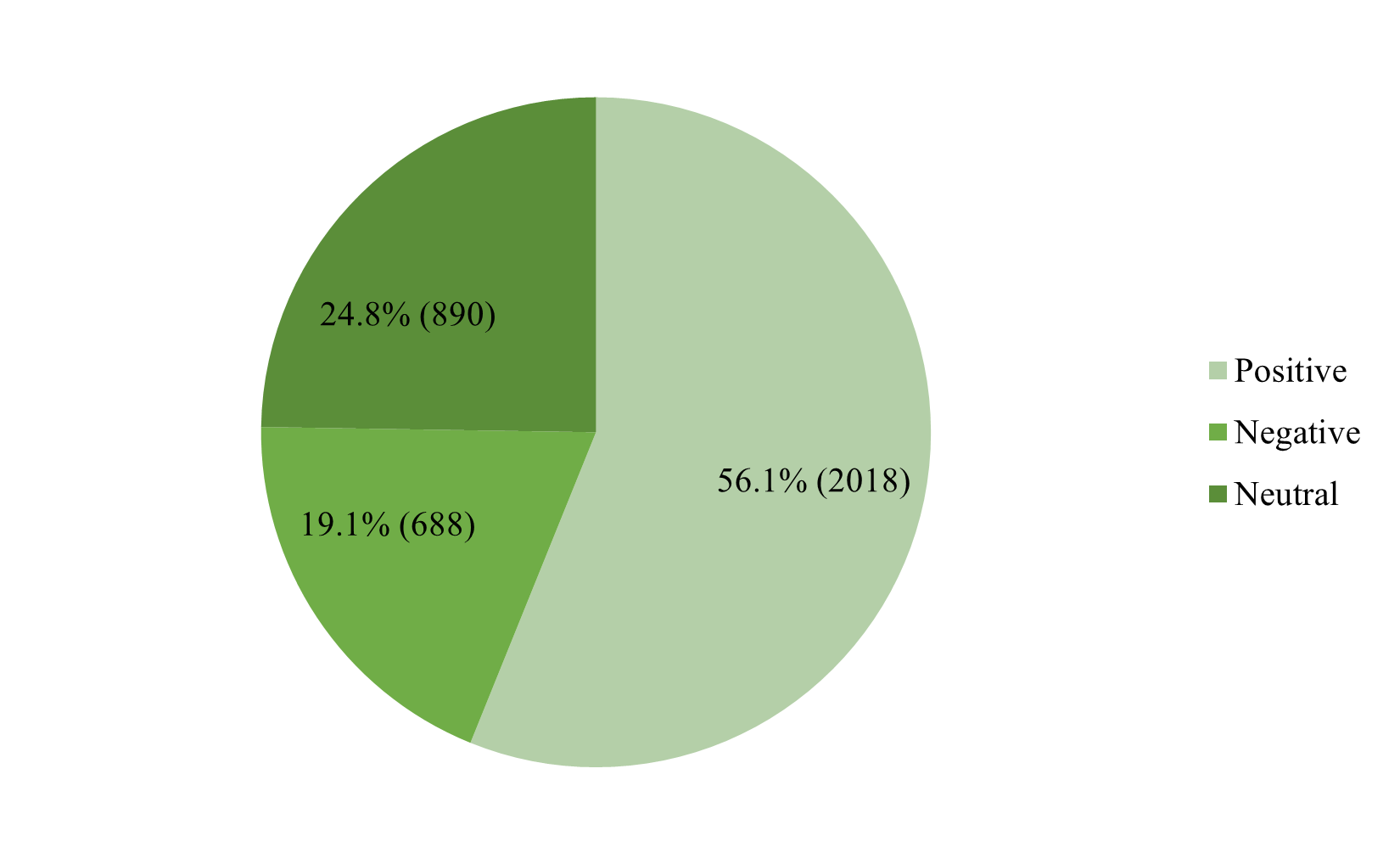

Supplement: Supplementary file 1 — Supplementary material [file mmc1.zip › Figure S4 Sentiment distribution (N=3,596).png]

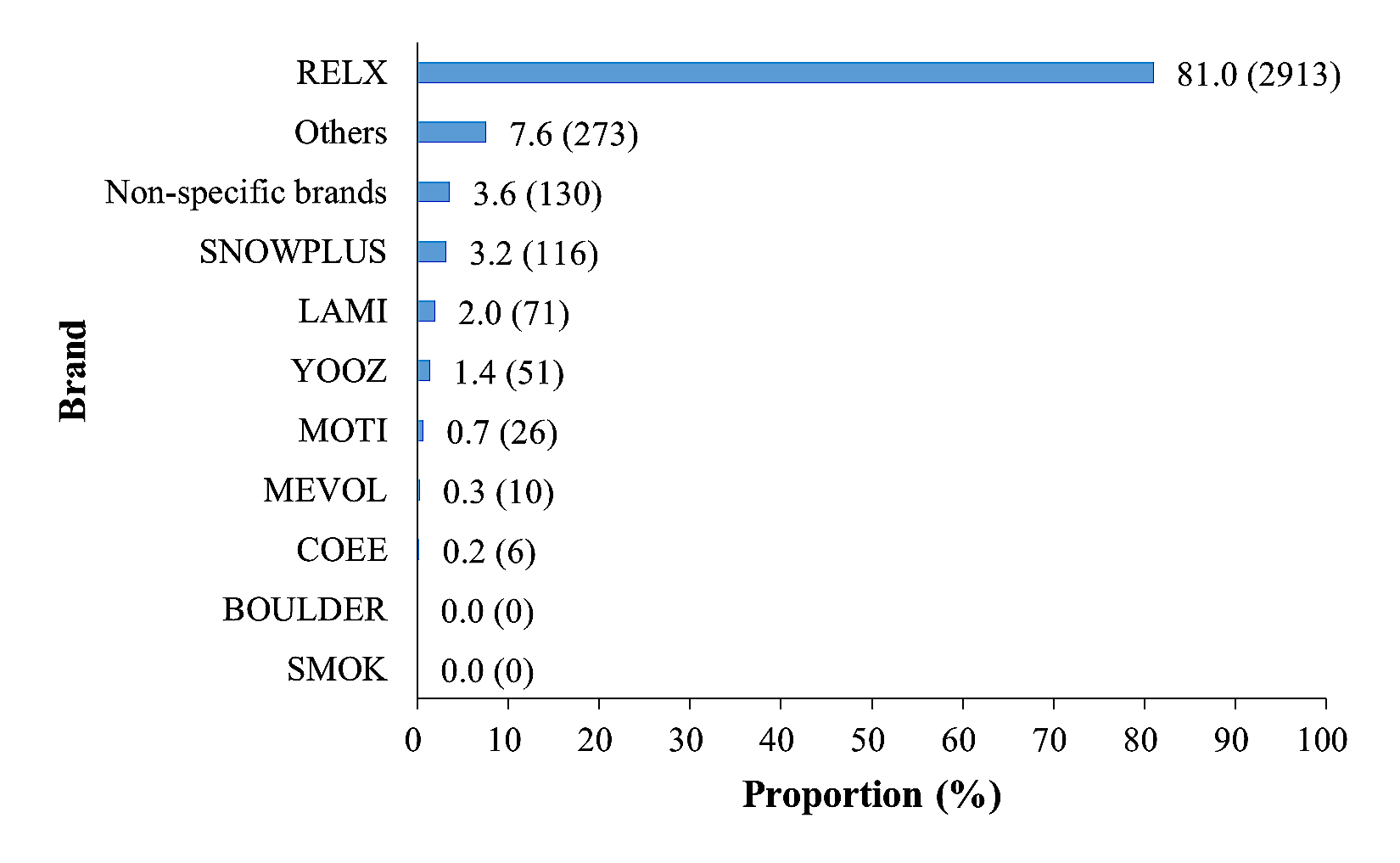

Supplement: Supplementary file 1 — Supplementary material [file mmc1.zip › Figure S2 Proportion of e-cigarette brand mentions (N=3,596).png]

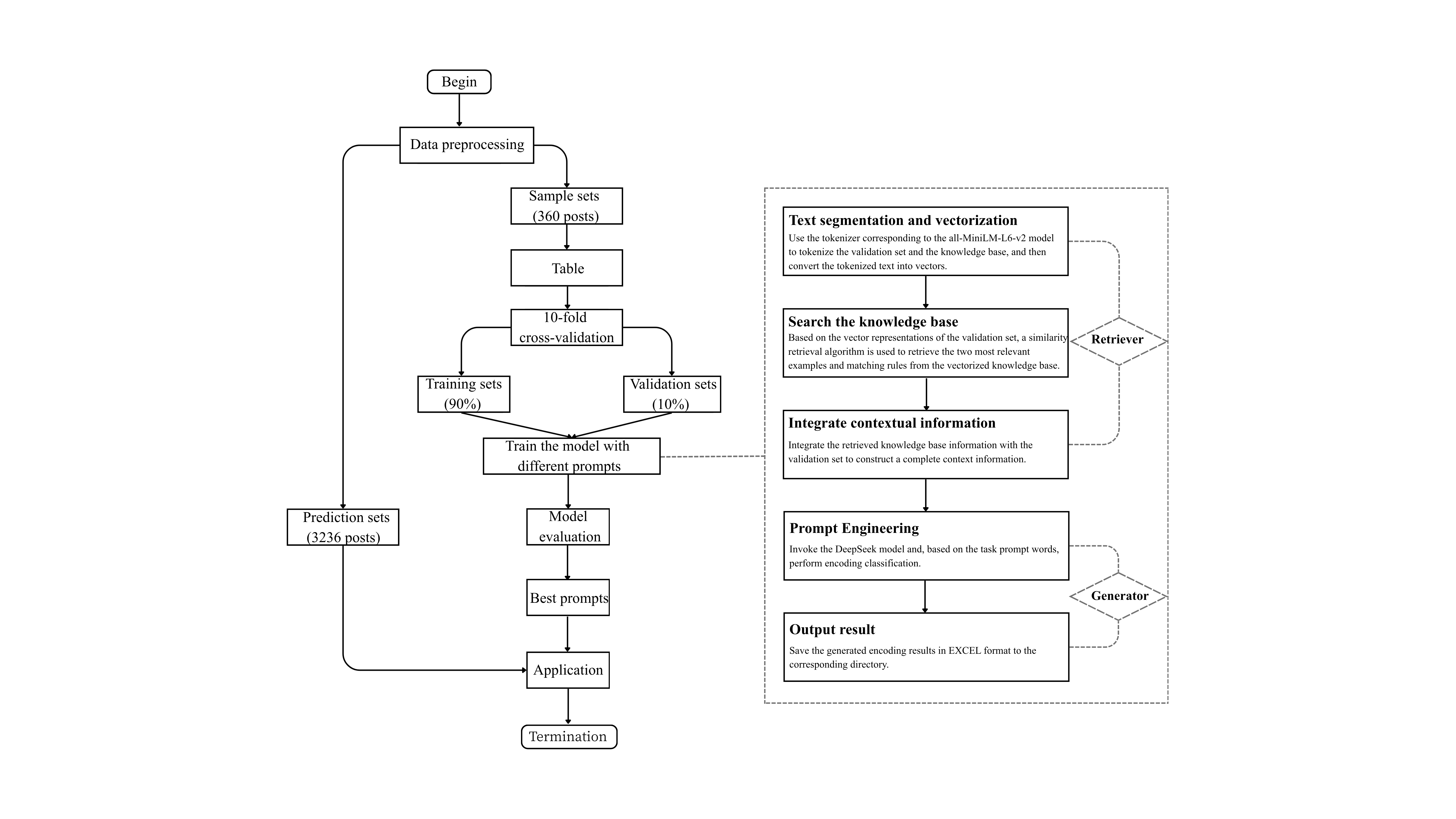

Supplement: Supplementary file 1 — Supplementary material [file mmc1.zip › Figure S1 Construction process of automated coding system for e-cigarette online marketing.png]
